# Monitoring Temporal Changes in SARS-CoV-2 Spike Antibody Levels and Variant-Specific Risk for Infection, Dominican Republic, March 2021–August 2022

**DOI:** 10.3201/eid2904.221628

**Published:** 2023-04

**Authors:** Eric J. Nilles, Michael de St. Aubin, Devan Dumas, William Duke, Marie Caroline Etienne, Gabriela Abdalla, Petr Jarolim, Timothy Oasan, Salome Garnier, Naomi Iihoshi, Beatriz Lopez, Lucia de la Cruz, Yosanly Cornelio Puello, Margaret Baldwin, Kathryn W. Roberts, Farah Peña, Kara Durski, Isaac Miguel Sanchez, Sarah M. Gunter, Alexander R. Kneubehl, Kristy O. Murray, Allison Lino, Sarah Strobel, Amado Alejandro Baez, Colleen L. Lau, Adam Kucharski, Emily Zielinski Gutiérrez, Ronald Skewes-Ramm, Marietta Vasquez, Cecilia Then Paulino

**Affiliations:** Brigham and Women’s Hospital, Boston, Massachusetts, USA (E.J. Nilles, M. de St. Aubin, D. Dumas, M.C. Etienne, G. Abdalla, P. Jarolim, T. Oasan, S. Garnier, N. Iihoshi, M. Baldwin, K.W. Roberts, K. Durski);; Harvard Humanitarian Initiative, Cambridge, Massachusetts, USA (E.J. Nilles, M. de St. Aubin, D. Dumas, S. Garnier, M. Baldwin, K.W. Roberts, K. Durski);; Harvard Medical School, Boston (E.J. Nilles, P. Jarolim);; Pedro Henríquez Ureña National University, Santo Domingo, Dominican Republic (W. Duke);; US Centers for Disease Control and Prevention, Central America Regional Office, Guatemala City, Guatemala (B. Lopez, E. Zielinski Gutiérrez);; Ministry of Health and Social Assistance, Santo Domingo (L. de la Cruz, Y. Cornelio Puello, F. Peña, I.M. Sanchez, R. Skewes-Ramm, C. Then Paulino);; Baylor College of Medicine and Texas Children’s Hospital, Houston, Texas, USA (S.M. Gunter, A.R. Kneubehl, K.O. Murray, A. Lino, S. Strobel);; Dominican Republic Office of the Presidency, Santo Domingo (A.A. Baez);; University of Queensland, Brisbane, Queensland, Australia (C.L. Lau);; London School of Hygiene and Tropical Medicine, London, England, UK (A. Kucharski);; Yale School of Medicine, New Haven, Connecticut, USA (M. Vasquez)

**Keywords:** COVID-19, coronavirus disease, SARS-CoV-2, severe acute respiratory syndrome coronavirus 2, viruses, respiratory infections, zoonoses, vaccine-preventable diseases, spike antibody, Dominican Republic

## Abstract

To assess changes in SARS-CoV-2 spike binding antibody prevalence in the Dominican Republic and implications for immunologic protection against variants of concern, we prospectively enrolled 2,300 patients with undifferentiated febrile illnesses in a study during March 2021–August 2022. We tested serum samples for spike antibodies and tested nasopharyngeal samples for acute SARS-CoV-2 infection using a reverse transcription PCR nucleic acid amplification test. Geometric mean spike antibody titers increased from 6.6 (95% CI 5.1–8.7) binding antibody units (BAU)/mL during March–June 2021 to 1,332 (95% CI 1,055–1,682) BAU/mL during May–August 2022. Multivariable binomial odds ratios for acute infection were 0.55 (95% CI 0.40–0.74), 0.38 (95% CI 0.27–0.55), and 0.27 (95% CI 0.18–0.40) for the second, third, and fourth versus the first anti-spike quartile; findings were similar by viral strain. Combining serologic and virologic screening might enable monitoring of discrete population immunologic markers and their implications for emergent variant transmission.

Given widespread unreported SARS-CoV-2 infections, variable immunologic response based on host immunogenicity, vaccine type, viral strain, timing and sequence of vaccine or viral exposure, and humoral waning, the global SARS-CoV-2 immune landscape is largely unknown. Most countries launched national COVID-19 vaccination campaigns during early 2021, but few studies have characterized population-level immunologic responses to SARS-CoV-2, and fewer have aimed to translate findings to immunologic protection. Many large national seroepidemiologic studies were conducted in the pre–COVID-19 vaccine era and before emerging variants of concern, focusing primarily on seroprevalence (i.e., the presence or absence of SARS-CoV-2 antibodies) but not antibody levels (*1*–*4*). This focus was largely because of an urgent need to understand population-level transmission and transmission risks, but it was also the result of limited understanding of what binding antibody levels mean for immunologic protection and whether quantification of binding antibodies translate into actionable or otherwise useful data. Although neutralizing antibodies are the generally accepted standard correlate of protection against symptomatic infection (*5*–*7*), measuring neutralizing activity is slow and resource intensive and therefore impractical for most population-based studies, particularly in low- and middle-income countries. Recent approaches have combined screening subsets of populations for neutralizing activity and applying machine learning methods to estimate population-level immunologic protection (*8*), but those approaches still require neutralization testing of a certain fraction of samples in addition to applying machine learning methods. The direct use of binding antibodies to estimate immunologic protection is, therefore, attractive, at least for population-based studies, where the tolerance for imprecision may be higher than vaccine efficacy trials. Although global health authorities including the World Health Organization previously cautioned against using binding antibodies to assess immunologic protection, several large studies subsequently demonstrated that SARS-CoV-2 spike binding antibodies (hereafter S antibodies) largely track with protection against infection (*5*–*7*). However, those studies were conducted in the setting of controlled vaccine efficacy studies and before emergence of highly immune evasive viral variants, so the utility of S antibodies for understanding immunologic protection in a real-world setting, in which transmission is driven by Omicron-derived strains, is unknown.

Given those knowledge gaps, which we believe are essential to address in order to inform and prioritize public health activities moving forward, we conducted a study using a novel methodologic approach to first characterize temporal changes in S antibody titers across a discrete population. In addition, we evaluated the utility of S antibodies for assessing risk for acute SARS-CoV-2 infection across viral variants and strains.

## Methods

### Setting

The Dominican Republic is an upper-middle-income Latin American country that shares the island of Hispaniola with Haiti. With ≈11 million residents, it is the second most populous country in the Caribbean (*9*,*10*). The first laboratory-confirmed case of SARS-CoV-2 infection was reported in the Dominican Republic on March 1, 2020, and strict public health measures commensurate with those in most countries of the region were implemented (*11*). Six discrete waves of SARS-CoV-2 transmission were observed during March 2020–August 2022; the last 3 waves were predominantly attributable to B.1.617.2 Delta (October–November 2021); BA.1 Omicron (January–February 2022); and post–BA.1 Omicron variants, including BA.2, BA.4, and BA.5 (June–August 2022). Peak national cases reported per day were 4–5 times higher during the BA.1 wave (≈6,000 cases/day) than during the other waves (≈1,100–1,300 cases/day) (*6*). A national COVID-19 vaccination campaign was launched in late February 2021, and by March 22, 2021 (the start of our study), ≈7.4% of the national population had received 1 COVID-19 vaccine dose (*12*). The principal COVID-19 vaccines administered were inactivated viral CoronaVac (Sinovac, https://www.sinovac.com), adenovirus vector ChAdOx1-S (Oxford/AstraZeneca, https://www.astrazeneca.com), and mRNA BNT162b2 (Pfizer/BioNTech, https://www.pfizer.com) vaccines. 

Latin America emerged as a global SARS-CoV-2 hotspot early in the COVID-19 pandemic; model estimates suggested that by November 2021 the regional cumulative population infected was 57.4% (95% CI 51.7%–63.1%) (*13*). A national cross-sectional household serologic survey in the Dominican Republic estimated that by August 2021, 85.0% (95% CI 82.1%–88.0%) of the >5-year-old population had been immunologically exposed through vaccination, infection, or both, and 77.5% (95% CI 71.3%–83.0%) had been previously infected (*8*).

### Study Design, Study Sites, and Participant Selection

We conducted prospective enrollment across 2 study sites: Hospital Dr. Antonio Musa, located in San Pedro de Macoris Province in the southeast of the country, and Dr. Toribio Bencosme Hospital in Espaillat Province in the northwest of the country. Those study sites are part of a longitudinal US Centers for Disease Control and Prevention (CDC)–funded acute febrile infection enhanced surveillance platform, which, in collaboration with the Ministry of Health and Social Assistance, aims to better characterize the epidemiology and transmission of acute febrile infection pathogens, including SARS-CoV-2. Patients >2 years of age who arrived at the study sites with an undifferentiated fever, either measured (>38.0°C) or by history, or with new onset anosmia or ageusia were invited to participate. Study staff (all of whom were medical doctors) conducted enrollment 5 days/week from 8 a.m. to 5 p.m. We administered questionnaires by using the KoBo Toolbox data collection platform (https://www.kobotoolbox.org) on electronic tablets to collect individual-level covariates, including demographic data (e.g., age, sex, race, and ethnicity); underlying medical conditions (e.g., hypertension, coronary heart disease, diabetes, active cancer, chronic kidney disease, stroke, asthma, and chronic obstructive pulmonary disease); weight and height; primary occupation; symptom onset date; and number, date, and type of COVID-19 vaccines received. We collected nasopharyngeal swab and venous blood samples from all participants at the time of enrollment. We processed blood as serum samples and stored biologic samples at −80°C.

To assess the association between S antibody levels at the time of SARS-CoV-2 diagnosis (peri-infection) and risk for SARS-CoV-2 infection, we used a test-negative approach that first assigned study participants into 2 groups based on SARS-CoV-2 virologic test result. We then assessed crude S antibody levels between groups and subsequently performed univariable and multivariable binomial logistic regression with S antibody levels categorized by quartile. We considered peri-infection antibody levels to reflect antibody levels at the time of infection.

### Ethical Considerations

We obtained written consent for all participants. For children <18 years of age, except emancipated minors, we obtained consent from the legal guardian. Written assent was provided by adolescents 14–17 years of age and verbal assent by children 7–13 years of age. The study was reviewed and approved by the National Council of Bioethics in Health, Santo Domingo (approval no. 013–2019), the Institutional Review Board of Pedro Henríquez Ureña National University, Santo Domingo, and the Massachusetts General Brigham Human Research Committee, Boston, Massachusetts, USA (approval no. 2019P000094). Study procedures and reporting adhere to STROBE criteria for observational studies.

### Immunoassay Characteristics

We measured serum pan-Ig against the SARS-CoV-2 S glycoprotein at Brigham and Women’s Hospital (Boston, Massachusetts, USA) on the Roche Elecsys SARS-CoV-2 electrochemiluminescence immunoassay that uses a recombinant protein–modified double-antigen sandwich format (Roche Diagnostics, https://www.roche.com). We calibrated the assay with positive (wild-type) and negative quality controls before analyses. We quantified values ranging from 0.40 to 250 U/mL representing the primary measurement range; we reported values <0.40 U/mL as 0.40 U/mL. Samples with measured values >250 U/mL underwent automated 1:50 dilution with further 1:10 dilution for samples >12,500 U/mL, representing an upper limit of detection of 125,000 U/mL. We considered samples to be reactive according to the manufacturer cutoff index (>0.8 U/mL). We report values as binding antibody units (BAU) that equal Elecsys SARS-CoV-2 S antibody U/mL in accordance with manufacturer’s recommendations and the World Health Organization’s international standard and international reference panel for SARS-CoV-2 Ig (*14*). Assay performance measures reported by a largest, non–manufacturer-sponsored study registered a specificity of 99.8% (95% CI 99.3%–100%) and sensitivity of 98.2% (95% CI 96.5%–99.2%) (*15*).

### Virologic Assays

We assessed acute SARS-CoV-2 infection by using a real-time reverse transcription PCR nucleic acid amplification test (NAAT) on nasopharyngeal specimens using the Allplex SARS-CoV-2 kit (Seegene, https://www.seegene.com), which amplifies the envelope, nucleocapsid, and RNA dependent RNA polymerase genes. Conditions for amplifications were 50°C for 20 min, 95°C for 15 min, followed by 45 cycles of 95°C for 10 s and 60°C for 15 s and 72°C for 10 s. We considered samples to be positive according to the manufacturer recommendations (i.e., with a cycle threshold value <37). We defined a cycle threshold value >38 as a negative. We performed genomic sequencing on a subset of NAAT-positive samples (Appendix).

### Classification and Statistical Analysis

We analyzed mean SARS-CoV-2 seroprevalence and number of COVID-19 vaccines received by 7-day intervals, starting on the first day of the study period. We defined viral strain transmission phases according to the predominant circulating viral strain based on genome sequencing of 237 SARS-CoV-2 NAAT–positive study samples: March 22, 2021–August 15, 2021 (pre-Delta), August 16, 2021–December 23, 2021 (Delta), December 24, 2021–April 30, 2022 (BA.1 [Omicron]), and May 1, 2022–August 17, 2022 (post-BA.1). Because phases varied in duration, we created a second date partition that captured largely similar 3- to 4-month time intervals: March–June 2021 (March 22–June 30, 2021), July–September 2021, October–December 2021, January–April 2022, and May–August 2022 (May 1–August 17, 2022). We analyzed data by date of participant enrollment. We calculated days post–symptom onset (DPSO) by subtracting the symptom onset date from the date of enrollment. For 10 participants without symptom onset date, we imputed DPSO as the DPSO mode for all other participants. We aggregated age into 3 groups (2–17, 18–54, and >55 years) and selected cutoffs to capture groups with documented differences in seroprevalence in the Dominican Republic while minimizing data sparsity among older adults. Because our study was an observational study of prospectively enrolled patients, we included all eligible participants with required data and performed no sample size power calculation.

We conducted analyses by using the R statistical programming language (R version 4.1.3) with finalfit (glm) for univariable and multivariable logistic regression (*16*). We performed data visualization by using visreg and ggplot2 (*17**,**18*).

### Data Sources

We obtained national SARS-CoV-2 cases, deaths, and vaccination data from the COVID-19 GitHub repository (*6*). We enumerated other data during the study.

## Results

**Figure 1:**
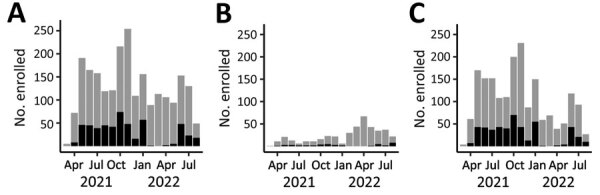
Number of participants (N = 2,300) enrolled per month, by age group, in a study of SARS-CoV-2 spike antibody levels, Dominican Republic, March 2021–August 2022. A) All ages; B) 2–17 years of age; C) >18 years of age. Gray bar sections indicates SARS-CoV-2 NAAT–negative participants; black bar sections indicate SARS-CoV-2 NAAT–positive participants. Labels on x-axis indicate complete months, except March 2021, which represents enrollment starting March 22, 2021, and August 2022, which represents enrollment through August 17, 2022.

During March 22, 2021–August 17, 2022, we invited 2,814 eligible patients to participate in our study, of whom 2,502 (89.0%) were enrolled. Of those, 2,300 (91.9%) had complete virologic, serologic, and demographic data and were included in analyses (Appendix Figure 1). The median age of participants was 31 years (interquartile range 7–55 years); 1,422/2,300 (61.8%) were women and girls (Table 1). The mean interval between symptom onset and enrollment was 4.0 days (mean absolute difference 2.5 days) for all participants and 3.7 days (mean absolute difference 2.1 days) for SARS-CoV-2 NAAT–positive participants (Appendix Table 1). Overall SARS-CoV-2 NAAT test positivity was 22.4% (517/2,300) (Figure 1). 

**Table 1:** Population characteristics of participants in study of SARS-CoV-2 spike antibody levels, by SARS-CoV-2 NAAT status, Dominican Republic, March 2021–August 2022*

Variable	NAAT-positive, n = 517	NAAT-negative, n = 1,783	Total, N = 2,300
Sex			
F	327 (63.2)	1,095 (61.4)	1,422 (61.8)
M	189 (36.6)	688 (38.6)	877 (38.1)
Median age (IQR), y	36 (10–62)	30 (5.5–54.5)	31 (7–55)
Age group, y			
2–17	43 (8.3)	376 (21.1)	419 (18.2)
18–54	368 (71.2)	1,189 (66.7)	1,557 (67.7)
>55	106 (20.5)	218 (12.2)	324 (14.1)
Area of residence			
Rural or semirural	366 (70.8)	1,245 (69.8)	1,611 (70.0)
Urban	147 (28.4)	514 (28.8)	661 (28.7)
Unclassified	4 (0.8)	24 (1.3)	28 (1.2)
No. household residents			
1–2	104 (20.1)	377 (21.1)	481 (20.9)
3–4	245 (47.4)	838 (47.0)	1,083 (47.1)
5–6	130 (25.1)	430 (24.1)	560 (24.4)
>7	38 (7.4)	135 (7.6)	173 (7.5)
Enrollment site			
San Pedro de Macoris Province	243 (47.0)	802 (45.0)	1,045 (45.4)
Espaillat Province	274 (53.0)	981 (55.0)	1,255 (54.6)
Underlying condition†			
Respiratory disease	52 (10.1)	245 (13.7)	297 (12.9)
Cardiovascular disease	105 (20.3)	277 (15.5)	382 (16.6)
Diabetes	44 (8.5)	114 (6.4)	158 (6.9)
BMI >30	89 (17.2)	332 (18.6)	421 (18.3)
Pregnancy	18 (3.5)	57 (3.2)	75 (3.3)
No. COVID-19 vaccine doses			
0	150 (29.0)	604 (33.9)	754 (32.8)
1	61 (11.8)	177 (9.9)	238 (10.3)
2	262 (50.7)	766 (43.0)	1,028 (44.7)
3	44 (8.5)	230 (12.9)	274 (11.9)
4	0 (0.0)	6 (0.3)	6 (0.3)
Study period interval			
Mar–Jun 2021	99 (19.1)	335 (18.8)	434 (18.9)
Jul–Sep 2021	126 (24.4)	271 (15.2)	397 (17.3)
Oct–Dec 2021	137 (26.5)	442 (24.8)	579 (25.2)
Jan–Apr 2022	61 (11.8)	403 (22.6)	464 (20.2)
May–Aug 2022	94 (18.2)	332 (18.6)	426 (18.5)

*Values are no. (%) except as indicated. Number of household residents missing for 3 participants. One participant reported ‘other’ for sex and was not included in analyses. BMI, body mass index; IQR, interquartile range; NAAT, nucleic acid amplification test. †Respiratory disease includes chronic obstructive pulmonary disease, asthma, other chronic respiratory diseases. Cardiovascular disease includes hypertension and coronary artery disease. BMI calculated by dividing weight in pounds by height in inches squared and multiplied by a conversion factor of 703.

### Changes in COVID-19 Vaccination Rates

**Figure 2:**
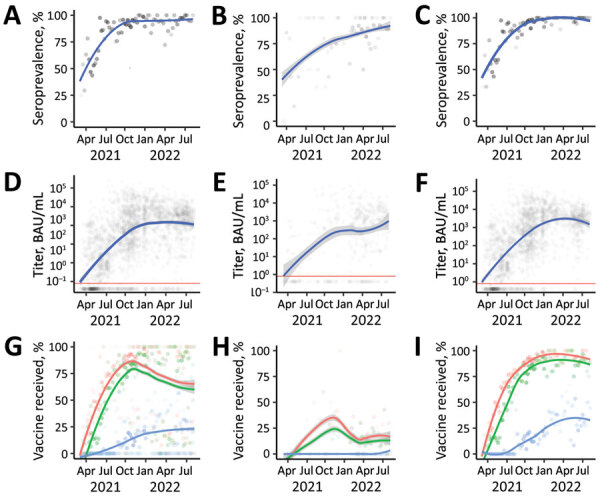
SARS-CoV-2 S antibody seroprevalence, titers, and vaccine doses of participants enrolled (N = 2,300) in a study of SARS-CoV-2 S antibody levels, by age group, Dominican Republic, March 2021–May 2022. A–C) Seroprevalence among study participants of all ages (A), 2–17 years of age (B), and >18 years of age (C). Gray dots indicate weekly mean values; increased dot intensity reflected more observations. Blue line indicates locally estimated scatterplot smoothing (LOESS) smoothed seroprevalence; gray shading indicates 95% CI around the smoothed estimate. D–F) Titers among study participants of all ages (D), 2–17 years of age (E), and >18 years of age (F), by week, plotted on a log scale. Each gray dot indicates a unique study participant (n = 1,910). Blue lines indicate LOESS smoothed antibody levels; gray shading indicates 95% CI around the smoothed estimate. Horizontal red line indicates manufacturer recommended cutoff index (>0.800 BAU /mL); values above the line represent a positive result and values below the line a negative result. G–I) Percentage of weekly enrolled participants of all ages (G), 2–17 years of age (H), and >18 years of age (I) who had received >1 (red dots), >2 (green dots), or >3 (blue dots) COVID-19 vaccine doses; increased dot intensity reflects more observations. Colored lines indicate LOESS smoothed percentage; gray shading indicates 95% CI around smoothed percentage. BAU, binding antibody units; S, spike.

After the launch of the national COVID-19 vaccination campaign in late February 2021, coverage among the study population increased rapidly, consistent with nationally reported data. By December 2021, approximately 75% of study participants had completed a 2-dose primary series (Figure 2). Unexpectedly, overall vaccination rates declined after December 2021, a finding attributable to an increase in younger pediatric patients who were ineligible for COVID-19 vaccines at the time, a finding consistent across study sites (Appendix Figure 2, 3). Vaccination coverage among adults remained high through the remainder of the study, and by June–August 2022, approximately 80% of adults had completed a primary vaccine series and about 35% had received a third vaccine dose. Vaccination coverage among adult study participants appeared to be modestly higher than national reported COVID-19 data (*7*), but without age-stratified national vaccination data, which were not available, we could not make direct comparisons.

### Temporal Changes in S Antibody Seropositivity and Levels

**Figure 3:**
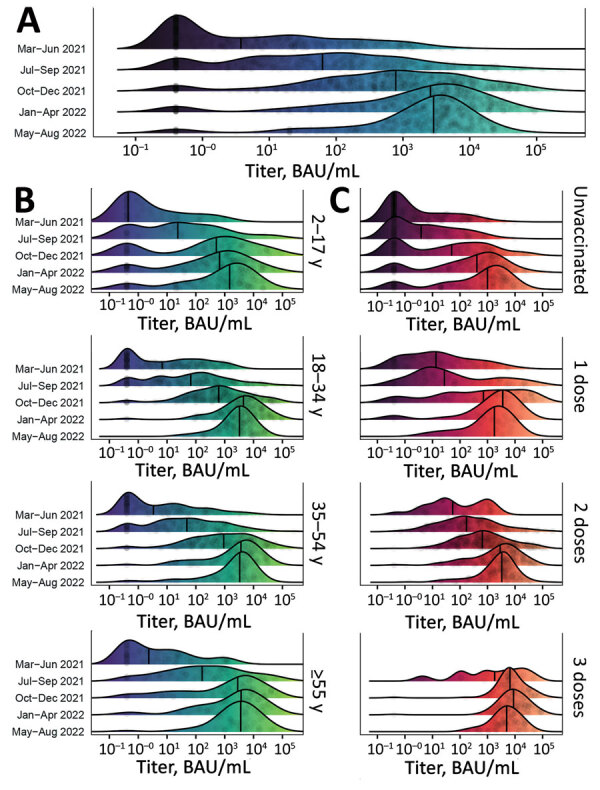
Distribution of SARS-CoV-2 S antibody titers among participants in a study of SARS-CoV-2 S antibody levels, Dominican Republic, March 2021–August 2022. A) Smoothed density plot demonstrates log-adjusted distribution of anti-S antibody titers among all study participants (N = 2,300), stratified by date interval when study participants were enrolled from earliest (March–June 2021, upper) to latest (May–August 2022, lower). Study interval labels indicate complete months except March 2021, which represents enrollment starting March 22, 2021, and August 2022, which represents enrollment through August 17, 2022. B) Smoothed density plot demonstrates log-adjusted distribution of S antibody titers among study participants (n = 2,300) stratified by age group. Dark purple shading indicates lower S titers and light green higher titers. C) Smoothed density plot demonstrates log-adjusted distribution of S antibody titers among participants (n = 2,293), stratified by number of COVID-19 vaccine doses received from none (unvaccinated, top plot) to 3 (bottom plot). Darker red shading indicates lower S titers and light orange higher titers. Six participants who received 4 COVID-19 vaccine doses not included. Values for 3 vaccine doses for March–June 2021 period plot not shown given sparsity of datapoints (n = 1). For all plots, gray circles represent titer adjusted individual study participant values. Narrow vertical black lines indicates median values. Lower limit of assay measurement is 0.4 BAU/mL, and values <0.4 BAU/mL are represented as 0.4 BAU/mL, with smoothing extending curves below the lower measurement limit. Therefore, density plot shading is used for illustrative purposes. Table 2 and Appendix Tables 2, 3) summarize data used for plots. BAU, binding antibody units; S, spike.

During the March–June 2021 and May–August 2022 study periods, the proportion of participants testing positive for S antibodies increased from 61.1% to 95.8%. Geometric mean titer (GMT) values increased 202-fold, median titer values increased 757-fold (Table 2; Figure 3, panel A), and near–log linear increases occurred across the study population through January 2022, when overall GMT flattened. We visualized the trend in overall antibody titers during the study period (Figure 3, panel A) and further stratified those trends by age group (Figure 3, panel B), and vaccination status (Figure 3, panel C). We observed progressive increases in S antibody titers over time across all age groups and within each vaccine dose category. For example, among recipients of 2 vaccine doses, GMT increased from 72.1 BAU/mL (95% CI 40.1–129.7 BAU/mL) during March–June 2021 to 2,153.2 BAU/mL (95% CI 1,684.7–2,752.1 BAU/mL) during May–August 2022; we observed similar trends across recipients of 1 vaccine dose (Figure 2, panel C; Appendix Table 3). We observed a less pronounced increase across recipients of 3 vaccine doses, who demonstrated high titers, measured on a logarithmic scale, across all study periods. Increases in GMT over time within vaccine dose categories probably represent ongoing immunologic exposure attributable to SARS-CoV-2 infections and transition from the less immunogenic Sinovac vaccine early in the national vaccination campaign to the mRNA BNT162b2 vaccine in late 2021. As evidenced by progressively increasing S antibody titers over time among unvaccinated study participants, and consistent with nationally reported data, substantial SARS-CoV-2 transmission continued through most of the study period. However, despite ongoing transmission, S antibody titers remained substantially lower in unvaccinated participants than in vaccinated participants. For example, during May–August 2022, GMTs among unvaccinated participants represented 25.7% of S antibody GMT among recipients of 1 vaccine dose, 13.1% among recipients of 2 vaccine doses, and 6.3% among recipients of 3 vaccine doses (Figure 2, panel C; Appendix Table 3).

**Table 2:** SARS-CoV-2 spike binding antibody serostatus, geometric mean titers, and median titers of participants in study of SARS-CoV-2 spike antibody levels, by study period interval, Dominican Republic, March 2021–August 2022*

Study period interval	No. patients	Seropositive, no. (%)†	GMT (95% CI)	Median titer, BAU/mL (Q1—Q3)
Mar–Jun 2021	434	265 (61.1)	6.6 (5.1–8.7)	3.8 (0.4–57.5)
Jul–Sep 2021	397	344 (86.6)	62.8 (45.8–86.0)	62.5 (6.0–581.8)
Oct–Dec 2021	579	543 (93.8)	559.4 (439.8–711.5)	781.7 (104.9–4,813.5)
Jan–Apr 2022	463	434 (93.7)	1,180.3 (906.3–1,537.2)	2,578 (390.8–8,137.5)
May–Aug 2022	427	409 (95.8)	1,332.4 (1,055.3–1,682.3)	2,876 (775.8–5,483.5)

*N = 2,300. Study periods indicate complete months except March 2021, which represents enrollment starting March 22, 2021, and August 2022, which represents enrollment through August 17, 2022. BAU, binding antibody units; GMT, geometric mean titer. †Seropositive defined as SARS-CoV-2 spike binding antibodies above the test manufacturer’s cutoff index (>0.8 BAU/mL).

### Association between S Antibody Titers and SARS-CoV-2 NAAT Status

Although we observed a substantial increase in S antibody levels across all demographic groups during the study period, the implications for immunologic protection were unclear. Therefore, we used a test-negative approach to assess whether simple unadjusted S antibody levels were associated with the NAAT test result. We identified a consistent inverse association across all phases of transmission (Table 3), observing broadly similar ratios when we compared S antibody levels between NAAT-positive and NAAT-negative participants across viral strains.

**Table 3:** Geometric mean and median spike binding antibody titers of participants in study of SARS-CoV-2 spike antibody levels, by SARS-CoV-2 NAAT status and phase of predominant circulating viral strain, Dominican Republic, March 2021–August 2022*

Phase†	SARS-COV-2 NAAT result	No. (%) participants	GMT, BAU/mL (95% CI)	Fold difference in GMT‡	Median titer, BAU/mL (Q1–Q3)	Fold difference in median titer
Pre-Delta	Negative	495 (76.6)	14.1 (10.9–18.2)	**3.4**	13.3 (0.8–132.8)	5.5
	Positive	151 (23.4)	4.1 (2.6–6.5)		2.4 (0.4–24.4)	
Delta	Negative	553 (72.4)	604.8 (475.3–769.7)	**3.9**	792.3 (127.8–4,805)	4.5
	Positive	211 (27.6)	154.7 (97.8–244.7)		176.2 (24.7–1,638.5)	
Omicron BA.1	Negative	403 (86.9)	1,288.3 (965.4–1,719.2)	**1.8**	2,822 (511.1–8,656)	3.4
	Positive	61 (13.1)	713.8 (375.1–1,358.5)		837.2 (126.8–5,739)	
Omicron BA.2/4/5	Negative	332 (77.9)	1,541.4 (1,183.4–2,007.6)	**2.0**	3,202 (1,011–6,173)	1.7
	Positive	94 (22.1)	759.6 (468.1–1,232.6)		1,835 (197.7–3,882.2)	
Total	Negative	1,783 (77.5)	300.6 (256.5–352.4)	**3.5**	725 (31.6–4,351.5)	6.0
	Positive	517 (22.5)	85.8 (62.9–117.1)		121.2 (4.6–1,905)	

*N = 2,300. BAU, binding antibody units; GMT, geometric mean titer; NAAT, nucleic acid amplification test. †Phases based on dominant circulating strain: March 22–August 15, 2021 (pre-Delta, primarily Mu, Gamma, Iota, and Lambda strains), August 16–December 23, 2021 (Delta), December 24, 2021–April 30, 2022 (Omicron, BA.1), and May 1–August 17, 2022 (Omicron, BA.2, BA.4, BA.5). Appendix Figure 5 shows sequence-confirmed variant by week. ‡Fold difference is GMT or median titer of NAAT-negative participants divided by the titer of NAAT-positive participants. Bolded type indicates statistical significant result (p<0.001, by t test for GMT difference between NAAT-negative and positive-study participants).

Using multivariable analyses, we again identified an inverse association between S antibody quartile and odds ratio (OR) for a positive NAAT; results demonstrated a clear biologic gradient (Table 4). Younger age (2–17 years) was associated with a lower OR (0.45 [95% CI 0.29–0.68]; p<0.001) and older age (>55 years) with higher OR (1.58 [95% CI 1.19–2.07]; p = 0.001) for a positive NAAT test compared with the 18–54 year age group; we observed similar but largely nonsignificant trends when these data were stratified by phase (Appendix Tables 4–8). We observed no consistent association between NAAT status and sex or number of COVID-19 vaccine doses received after controlling for S antibody levels (Appendix Tables 4–8).

**Table 4:** Multivariable odds ratios for a SARS-CoV-2 NAAT-positive test result in participants in study of SARS-CoV-2 spike antibody levels, by phase of predominant circulating viral strain, Dominican Republic, March 2021–August 2022*

S antibody titer quartile	OR (95% CI)†				
	Total, N = 2,300	Pre-Delta, n = 646	Delta, n = 764	Omicron (BA.1), n = 464	Omicron (BA.2/4/5), n = 426
Q1	Referent	Referent	Referent	Referent	Referent
Q2	0.55 (0.40–0.74)§	0.25 (0.14–0.44)§	0.38 (0.23–0.62)§	0.69 (0.27–1.76)	0.60 (0.30–1.17)
Q3	0.38 (0.27–0.55)§	0.13 (0.07–0.25)§	0.31 (0.19–0.51)§	0.46 (0.16–1.27)	0.30 (0.14–0.60)¶
Q4	0.27 (0.18–0.40)§	0.13 (0.06–0.25)§	0.31 (0.18–0.54)§	0.14 (0.04–0.42)¶	0.22 (0.09–0.50)§

*Phases based on dominant circulating strain: March 22–August 15, 2021 (pre-Delta, primarily Mu, Gamma, Iota, and Lambda strains), August 16–December 23, 2021 (Delta), December 24, 2021–April 30, 2022 (Omicron, BA.1), and May 1–August 17, 2022 (Omicron, BA.2, BA.4, BA.5). Anti-S, SARS-CoV-2 spike binding antibody. †Odds ratios with 95% CIs calculated using binomial multivariable logistic regression models with data presented for log-adjusted anti-S titers stratified by quartile. Quartiles calculated using the quantile function in R (*16*) and are specific to each phase. Appendix Table 10 shows phase and quartile anti-S geometric mean titers and median titers Model covariates include, in addition to anti-S titer quartile, number of COVID-19 vaccine doses received, days since last COVID-19 vaccine dose, sex, age, and month of sample collection. Appendix Tables 4–8 show full univariable and multivariable models, underlying data, predictor variables, and model performance measures. §p<0.001. ¶p<0.01.

We examined whether antibody levels trended higher based on the number of days between symptom onset and sample collection but were unable to detect a clear trend, even after stratifying by number of vaccine doses received (Appendix Figure 4), potentially because 50% of NAAT-positive case-patients had samples collected within 3 days of symptom onset and 90% within 6 days (Appendix Table 1). We also performed sensitivity analyses comparing samples collected 0–4 DPSO versus >5 DPSO and observed broadly similar findings, although the biologic gradient observed for samples collected 0–4 DPSO was less clearly defined for samples collected >5 DPSO (Appendix Table 9).

## Discussion

We report on the temporal change in SARS-CoV-2 S antibody prevalence levels over 18 months among patients enrolled through a longitudinal acute febrile illness surveillance platform in the Dominican Republic. The study period aligned with the beginning of the national COVID-19 vaccination campaign in late February 2021, providing a unique opportunity to characterize the evolution of S antibody levels in this setting and across a population that was largely COVID-19 vaccine–naive early in the study period. We observed a progressive increase in S antibody seroprevalence (from 61% to 96%), reflecting vaccination, infection, or both. Strikingly, during the study period, GMT increased ≈200-fold, and median titers increased 760-fold. To determine the implications of those findings for public health, we used a test-negative approach to assess antibody levels between NAAT-positive and NAAT-negative case-patients. We identified a consistent inverse association between S antibody titers and the likelihood of testing positive for SARS-CoV-2 by NAAT and extended those findings to phases of predominantly pre-Delta, Delta, Omicron BA.1, and Omicron-derivative strain waves of transmission.

S antibody levels were lower among those who tested positive versus negative for acute SARS-CoV-2 infection, a trend that was consistent across strains and after adjustment for potential confounders. When compared with the first quartile, the likelihood of testing positive was reduced by ≈45% for the second quartile, ≈60% for the third quartile and ≈75% for the fourth quartile. This finding aligns with several correlates of protection studies that reported S antibody levels track with risk for SARS-CoV-2 infection (*5*–*7*,*19*). We built on the findings from those prior studies, which were conducted before widespread transmission of variants of concern, to include transmission waves that were primarily driven by Delta, Omicron BA.1 and subsequent Omicron strains (BA.2, BA.4, and BA.5), and documented similar predictive utility of S antibody levels against those strains. These findings suggest that binding antibodies, at least total S antibody levels as measured in this study, track with functional measures of immunologic protection, such as ﻿viral neutralization, Fc-function, and potentially T-cell responses, as previously reported (*19*–*21*). Our findings suggest that, although total S antibody levels are probably inappropriate for adjudicating vaccine efficacy and vaccine approval, they are reasonable surrogate markers of immunologic protection against infection, including infection by emerging strains with substantial immune-evasion capacity. Given the relative simplicity, high-throughput capacity, and cost-effectiveness of measuring S antibody versus live or pseudoviral neutralizing activity, this approach may be suitable for characterizing population-level immunologic protection, creating transmission and prediction models, and informing national and regional public health policy.

For our analyses, we assumed a priori that S antibody levels measured at the time that a patient seeks care is a reasonable surrogate measure of levels at the time of infection, including among NAAT-positive case-patients, an approach that is not well-characterized but has been previously described (*22*). Most of the SARS-CoV-2 NAAT–positive case-patients can be assumed to have been mounting a humoral response to the acute infection at the time of sample collection, and persons previously exposed to SARS-CoV-2 antigens would be expected to mount a more rapid and robust anamnestic response. However, whether this response would obscure differences in antibody levels between groups, if present, was unclear before our study. Although we did identify a clear difference in risk for testing positive by S antibody levels, our observed differences in levels by NAAT status were probably attenuated.

Among the strengths of our study is that dedicated study staff prospectively enrolled study participants using well-defined procedures, administered standardized survey questionnaires, and simultaneously collected respiratory and blood samples. Enrollment of eligible patients was high (89%) for this type of surveillance study. Serum samples were tested with a widely used and validated immunoassay, and antibody titers were reported as internationally standardized units, so our findings can be compared across other settings. We developed an approach to understand temporal changes in population-level SARS-CoV-2 antibodies, methods that may be applicable to other settings. We performed genomic sequencing of a relatively large number of samples from among the current study population, and therefore were able to characterize timing of predominant SARS-CoV-2 strain transmission. Furthermore, we enrolled participants across geographically discrete settings, limiting the potential for study-site specific biases, and producing findings consistent across sites (Appendix Figure 2–3). 

The first limitation of our study is that ≈8% of enrolled study participants did not have serologic or NAAT data and were excluded from analyses, but the demographic profile of those persons largely reflected the final study population. Second, demographic information, underlying conditions, and COVID-19 vaccination status were self-reported, which may introduce recall or social-desirability biases, potentially affecting our findings in either direction. Third, we used a total S antibody immunoassay, and findings may be different for other assays that measure binding or neutralizing antibodies. Fourth, the immunoassay used in our study was designed to measure antibodies against the wild-type SARS-CoV-2 virus, and quantitative antibody measures may be different for highly immune-evasive variants (*23*). Fifth, sensitivity of NAAT for the detection of acute symptomatic SARS-CoV-2 infection is estimated to be 70%–95% (*24*); therefore, some infections may have been misclassified as noninfections, which would attenuate the differences in S antibody levels between cases and noncases reported in this study. Sixth, the study was conducted among a discrete population of patients seeking healthcare for undifferentiated fever; therefore, changes in antibody levels may not reflect the broader population, which would limit generalizability of our findings. However, as previously stated, findings were similar across our 2 geographically discrete study sites, suggesting that our findings are comparable across similar healthcare settings in the country.

In summary, we believe there are 3 broad findings from this study. First, we provide documentation of longitudinal changes in SARS-CoV-2 antibody titers after the launch of a national vaccination campaign. Second, we document that total S antibody levels track closely with risk for infection across multiple viral strains, including strains with highly effective immune-evasion capacity, suggesting that this test-negative approach may be valuable to model correlates of protection, while noting potential limitations as described previously. Third, we present a novel approach to monitoring changes in immune biomarkers among discrete populations, an approach that is relatively simple and can leverage existing surveillance infrastructure. Because future SARS-CoV-2 variants of concern and other emerging pathogens will occur, establishing pragmatic and sustainable methods to estimate population immune markers while simultaneously assessing strain-specific risks for infection may prove a valuable complement to existing surveillance infrastructure.

Appendix Additional information about monitoring temporal changes in SARS-CoV-2 spike antibody levels and variant-specific risk for infection, Dominican Republic, March 2021–August 2022.
