# Supplementary material for: Monitoring Temporal Changes in SARS-CoV-2 Spike Antibody Levels and Variant-Specific Risk for Infection, Dominican Republic, March 2021–August 2022
Source: Emerg Infect Dis. 2023 Apr;29(4):723–33. doi: 10.3201/eid2904.221628 (PMC10045678; doi:10.3201/eid2904.221628)
Supplement: Appendix — Additional information about monitoring temporal changes in SARS-CoV-2 spike antibody levels and variant-specific risk for infection, Dominican Republic, March 2021–August 2022. [file 22-1628-Techapp-s1.pdf]

Article DOI: https://doi.org/10.3201/eid2904.221628

Monitoring Temporal Changes in SARS-CoV-2 Spike Antibody Levels and Variant-Specific Risk for Infection, Dominican Republic, March 2021–August 2022

Appendix

Methods

Viral Genome Sequencing and Analysis

A total of 201 study samples were sequenced with analysis performed using the ARTIC pipeline and Oxford Nanopore Technologies tools (J.R. Tyson et al., unpub. data, https://doi.org/10.1101/2020.09.04.283077). Samples were randomly selected from NAAT positive samples from April 2021 to February 2022. Total nucleic acids (TNA) were extracted from nasopharyngeal samples using Zymo Research Quick DNA/RNA pathogen MiniPrep (Zymo Research Corp. Irvine, CA). Samples were initially screened by NAAT targeting the Sars-CoV-2 E gene as previously described (*1*). Any sample with a Ct-value ≤25 was sequenced to determine Sars-CoV-2 variant. SARS-CoV-2 genome amplification was performed on the TNA samples using the NEBNext ARTIC SARS-CoV-2 Companion Kit for Oxford Nanopore Technologies (New England Biolabs, Ipswich, USA) using the ARTIC V3 primers following manufacturer's instructions. The amplicons were sequenced using the SQK-LSK109 and EXP-NBD196 library prep kits with the Mk1B sequencer and R9.4.1 flow cell (Oxford Nanopore Technologies, Oxford, UK) following manufacturer's protocols. The sequence data were base-called using Guppy v6.1.5 with the high accuracy (HAC) model with barcodes required on both ends of the amplicons. The data were analyzed using the EPI2ME labs ARTIC workflow v0.3.18 (Oxford Nanopore Technologies, Oxford, UK) with default settings, which used Nextclade v1.11.0, and Pangolin v4.1.1 for clade and lineage classification (*2*–*4*). Samples which were missing more than three kilobases of genome sequence were not considered for analysis.

An additional 36 samples were sequenced using the Illumina DRAGEN COVID Lineage 3.5.11. Samples were randomly selected from NAAT positive samples from March to August 2022. TNA were extracted from nasopharyngeal samples using automatic magnetic extraction method with the RADI EXTRACTOR 192 and the RADI 192 viral DNA/RNA extraction kit (KH Medical, South Korea). Samples were screened using real-time reverse transcription polymerase chain reaction nucleic acid amplification tests (NAAT) of nasopharyngeal specimens using the Allplex SARS- CoV-2 kit (Seegene, Seoul, South Korea) that amplifies the E, N and the RdRP genes. Samples with a Ct-value ≤25 were sequenced. Library preparation was performed using the Illumina Covid Seq assay with the ARTIC V3 primers and were sequenced with Illumina´s MiSeq reagent kit V2, 300 cycles according to manufacturer specifications. Sequencing data was analyzed with the DRAGEN COVID Lineage 3.5.11 application on Illumina´s Basepace sequence hub with the default settings which used Nextclade 2.6.0 and Pangolin 4.1.2 pangolin-data 1.14 for clade and lineage classification.

References

1. Corman VM, Landt O, Kaiser M, Molenkamp R, Meijer A, Chu DK, et al. Detection of 2019 novel coronavirus (2019-nCoV) by real-time RT-PCR. Euro Surveill. 2020;25:2000045. PubMed https://doi.org/10.2807/1560-7917.ES.2020.25.3.2000045

2. Aksamentov I, Roemer C, Hodcroft E, Neher R. Nextclade: clade assignment, mutation calling and quality control for viral genomes. J Open Source Softw. 2021;6:3773. https://doi.org/10.21105/joss.03773

3. Rambaut A, Holmes EC, O'Toole Á, Hill V, McCrone JT, Ruis C, et al. A dynamic nomenclature proposal for SARS-CoV-2 lineages to assist genomic epidemiology. Nat Microbiol. 2020;5:1403–7. PubMed https://doi.org/10.1038/s41564-020-0770-5

4. O'Toole Á, Scher E, Underwood A, Jackson B, Hill V, McCrone JT, et al. Assignment of epidemiological lineages in an emerging pandemic using the pangolin tool. Virus Evol. 2021;7:b064. PubMed https://doi.org/10.1093/ve/veab064

Appendix Table 1. Days from symptom onset to enrollment by SARS-CoV-2 NAAT status

NAAT Status	n	Mean, days	Gmd	25%	50%	75%	90%	95%	Range, days
Positive	517	3.7	2.1	2	3	5	6	7	1–14
Negative	1783	4.1	2.6	2	4	5	7	8	0–20
All	2300	4	2.5	2	4	5	7	8	0–20

Blood collected for anti-S serologic tested at the time of enrollment. Gmd = Gini's mean difference. Percent represents the 25th, 50th, 75th, 90th, and 95th quantiles, with the 50th quantile representing the median number of days from symptom onset to enrollment/sample collection.

Appendix Table 2. Geometric mean and median anti-S antibody titers by age group and study period, Dominican Republic, March 2021 – August 2022

Age, years	Study period	N	Geometric mean (95% CI)	Median titer, BAU/ml (Q1, Q3)
2–17	Mar-Jun 2021	46	2.7 (1.3, 5.8)	0.4 (0.4, 14.5)
	Jul-Sep 2021	28	29.3 (7.6, 112.9)	24.4 (0.8, 254.6)
	Oct-Dec 2021	61	193.7 (69.6, 539.5)	510.4 (11.6, 3548)
	Jan-Apr 2022	146	260.4 (149.1, 454.7)	663.6 (43.2, 2743)
	May-Aug 2022	138	481.6 (281.8, 822.9)	1481.5 (96.8, 4115)
18–54	Mar-Jun 2021	315	8.1 (5.8, 11.1)	5.6 (0.4, 80.2)
	Jul-Sep 2021	297	51.9 (36.5, 73.9)	60.2 (5.4, 475.5)
	Oct-Dec 2021	450	613.2 (478.2, 786.3)	690 (114.2, 4307.5)
	Jan-Apr 2022	259	2598.2 (1983.4, 3403.6)	4084 (1250.5, 9794.5)
	May-Aug 2022	236	2105.1 (1673.9, 2647.2)	3286.5 (1234.2, 5793.5)
≥55	Mar-Jun 2021	73	5 (2.7, 9.4)	2.3 (0.4, 22.6)
	Jul-Sep 2021	72	185.9 (86.4, 399.7)	164.8 (19.8, 1615.8)
	Oct-Dec 2021	68	788.6 (351.9, 1767.6)	2824 (78.3, 7154.8)
	Jan-Apr 2022	58	1563.8 (762.4, 3207.7)	3694.5 (346.5, 8733.5)
	May-Aug 2022	53	2460.4 (1415.9, 4275.4)	3579 (1019, 6951)

GMT = geometric mean titer. Study periods indicate complete months except March 2021, which represents participants enrolled from 22 March 2021, and August 2022, which represents enrollment through 17 August 2022.

Appendix Table 3. Geometric mean and median anti-S antibody titers by number of vaccine doses and study period, Dominican Republic, March 2021 - August 2022

No. COVID-19 vaccine doses	Study period	N	Geometric mean (95% CI)	Median titer, BAU/ml (Q1, Q3)
None	Mar-Jun 2021	281	2.8 (2.1, 3.8)	0.4 (0.4, 20.6)
	Jul-Sep 2021	118	5.5 (3.4, 8.9)	3.9 (0.4, 37.5)
	Oct-Dec 2021	87	27.9 (13, 59.8)	49.9 (0.4, 606)
	Jan-Apr 2022	139	153.2 (88.2, 266.3)	411.8 (34.9, 1585)
	May-Aug 2022	129	282.5 (160.7, 496.6)	1000 (52, 3132)
One	Mar-Jun 2021	89	17.9 (10, 32.2)	13.7 (1.8, 115)
	Jul-Sep 2021	55	62.8 (24.8, 159.1)	27.1 (5.4, 1125.1)
	Oct-Dec 2021	48	399.6 (135.6, 1177.9)	712.4 (33.4, 7489.8)
	Jan-Apr 2022	26	1216 (395.9, 3734.7)	3565.5 (785.4, 5005)
	May-Aug 2022	22	1099.8 (458.7, 2636.7)	1784.5 (701.3, 4120)
Two	Mar-Jun 2021	64	72.1 (40.1, 129.7)	56.4 (15.4, 631.4)
	Jul-Sep 2021	214	209.4 (148.6, 295.1)	172.6 (38.1, 1051.5)
	Oct-Dec 2021	367	703.6 (560.9, 882.6)	649.2 (153.8, 2857)
	Jan-Apr 2022	202	1891.1 (1394.9, 2563.7)	2877 (666.6, 8203.2)
	May-Aug 2022	180	2153.2 (1684.7, 2752.1)	3302.5 (1225.5, 5419.2)
Three	Mar-Jun 2021	0	*NC*	*NC*
	Jul-Sep 2021	10	1277.8 (161.6, 10100.2)	2231.1 (277.6, 13452.2)
	Oct-Dec 2021	76	6844.5 (4775.2, 9810.6)	6532.5 (4523.5, 11250.5)
	Jan-Apr 2022	95	8209.3 (6044.9, 11148.6)	8844 (4483, 19871.5)
	May-Aug 2022	92	4485.5 (3477.5, 5785.7)	4950 (2290, 10284.5)
Four	Mar-Jun 2021	0	*NC*	*NC*
	Jul-Sep 2021	0	*NC*	*NC*
	Oct-Dec 2021	1	7782 (*NC, NC*)	7782 (*NC, NC)*
	Jan-Apr 2022	1	41355 (*NC, NC*)	41355 (*NC, NC*)
	May-Aug 2022	4	6377.9 (1951.7, 20841.9)	6589 (5293.2, 9048)

GMT = geometric mean titer. NC = not calculated. Study periods indicate complete months except March 2021, which represents participants enrolled from 22 March 2021, and August 2022, which represents enrollment through 17 August 2022.

Appendix Table 4. Univariable and multivariable odds ratios for factors associated with a positive SARS-CoV-2 virological test — Dominican Republic, 22 March 2021 – 17 August 2022

Variable	NAAT Negative, n (%)	NAAT Positive, n (%)	Univariate odds ratio	Multivariable odds ratio
Sex*				
Female	1095 (77.0)	327 (23.0)	*Ref*	*Ref*
Male	688 (78.4)	189 (21.6)	0.92 (0.75–1.13, p = 0.420)	0.93 (0.75–1.15, p = 0.494)
Age cat, years				
2–17	376 (89.7)	43 (10.3)	0.37 (0.26–0.51, p < 0.001)	0.46 (0.30–0.70, p < 0.001)
18–54	1189 (76.4)	368 (23.6)	*Ref*	*Ref*
≥55	218 (67.3)	106 (32.7)	1.57 (1.21–2.03, p = 0.001)	1.58 (1.20–2.08, p = 0.001)
Vaccine doses				
None	604 (80.1)	150 (19.9)	*Ref*	*Ref*
One	177 (74.4)	61 (25.6)	1.39 (0.98–1.95, p = 0.060)	1.04 (0.71–1.54, p = 0.827)
Two	766 (74.5)	262 (25.5)	1.38 (1.10–1.73, p = 0.006)	1.09 (0.75–1.58, p = 0.648)
Three	230 (83.9)	44 (16.1)	0.77 (0.53–1.11, p = 0.165)	0.82 (0.50–1.35, p = 0.441)
Four	6 (100.0)	0 (0.0)	*NC*	*NC*
Anti-S titer (quantile)				
Q1	399 (69.4)	176 (30.6)	*Ref*	*Ref*
Q2	431 (75.0)	144 (25.0)	0.76 (0.58–0.98, p = 0.035)	0.56 (0.41–0.76, p < 0.001)
Q3	463 (80.5)	112 (19.5)	0.55 (0.42–0.72, p < 0.001)	0.39 (0.27–0.56, p < 0.001)
Q4	490 (85.2)	85 (14.8)	0.39 (0.29–0.52, p < 0.001)	0.27 (0.18–0.40, p < 0.001)

Number in dataframe = 2300, Number in model = 2300, Missing = 0, AIC = 2254.3, C-statistic = 0.703, H&L = Chi-sq(8) 4.90 (p = 0.768). * One sex reported as *other* not presented in table.

Appendix Table 5. Univariable and multivariable odds ratios for factors associated with a positive SARS-CoV-2 virological test — Pre-Delta phase of transmission, Dominican Republic, 22 March to 15 August 2021

Variable	NAAT Negative, n (%)	NAAT Positive, n (%)	Univariate odds ratio	Multivariable odds ratio
Sex				
Female	301 (77.8)	86 (22.2)	*Ref*	*Ref*
Male	194 (74.9)	65 (25.1)	1.17 (0.81–1.69, p = 0.398)	1.12 (0.75–1.66, p = 0.571)
Age cat, years				
2–17	50 (84.7)	9 (15.3)	0.65 (0.29–1.29, p = 0.247)	0.59 (0.25–1.24, p = 0.185)
18–54	380 (78.2)	106 (21.8)	*Ref*	*Ref*
≥55	65 (64.4)	36 (35.6)	1.99 (1.24–3.13, p = 0.004)	2.14 (1.30–3.51, p = 0.003)
Vaccine doses				
None	266 (75.8)	85 (24.2)	*Ref*	*Ref*
One	98 (76.6)	30 (23.4)	0.96 (0.59–1.53, p = 0.860)	1.12 (0.61–2.07, p = 0.708)
Two	131 (78.4)	36 (21.6)	0.86 (0.55–1.33, p = 0.504)	1.21 (0.58–2.46, p = 0.610)
Three	0	0	*NC*	*NC*
Four	0	0	*NC*	*NC*
Anti–S titer (quantile)				
Q1	105 (64.8)	57 (35.2)	*Ref*	*Ref*
Q2	116 (71.6)	46 (28.4)	0.73 (0.46–1.17, p = 0.190)	0.45 (0.25–0.79, p = 0.006)
Q3	137 (85.1)	24 (14.9)	0.32 (0.19–0.55, p < 0.001)	0.19 (0.09–0.36, p < 0.001)
Q4	137 (85.1)	24 (14.9)	0.32 (0.19–0.55, p < 0.001)	0.19 (0.09–0.36, p < 0.001)

Number in dataframe = 646, Number in model = 646, Missing = 0, AIC = 669.4, C-statistic = 0.709, H&L = Chi-sq(8) 6.51 (p = 0.591).

Appendix Table 6. Univariable and multivariable odds ratios for factors associated with a positive SARS-CoV-2 virological test — Delta phase of transmission, Dominican Republic, 16 August 2021 to 23 December 2021

Variable	NAAT Negative, n (%)	NAAT Positive, n (%)	Univariate odds ratio	Multivariable odds ratio
Sex*				
Female	348 (73.1)	128 (26.9)	*Ref*	*Ref*
Male	205 (71.4)	82 (28.6)	1.09 (0.78–1.51, p = 0.615)	1.08 (0.76–1.53, p = 0.673)
Age cat, years				
2–17	60 (78.9)	16 (21.1)	0.72 (0.39–1.26, p = 0.264)	0.87 (0.43–1.68, p = 0.675)
18–54	420 (72.9)	156 (27.1)	*Ref*	*Ref*
≥55	73 (65.2)	39 (34.8)	1.44 (0.93–2.20, p = 0.098)	1.35 (0.83–2.18, p = 0.223)
Vaccine doses				
None	94 (69.6)	41 (30.4)	*Ref*	*Ref*
One	39 (61.9)	24 (38.1)	1.41 (0.75–2.64, p = 0.282)	2.06 (0.96–4.40, p = 0.063)
Two	342 (71.4)	137 (28.6)	0.92 (0.61–1.40, p = 0.689)	1.82 (0.92–3.59, p = 0.085)
Three	77 (89.5)	9 (10.5)	0.27 (0.12–0.56, p = 0.001)	0.64 (0.24–1.61, p = 0.353)
Four	1 (100.0)	0 (0.0)	*NC*	*NC*
Anti–S titer (quantile)				
Q1	104 (54.5)	87 (45.5)	*Ref*	*Ref*
Q2	142 (74.3)	49 (25.7)	0.41 (0.27–0.63, p < 0.001)	0.38 (0.23–0.62, p < 0.001)
Q3	152 (79.6)	39 (20.4)	0.31 (0.19–0.48, p < 0.001)	0.31 (0.19–0.51, p < 0.001)
Q4	155 (81.2)	36 (18.8)	0.28 (0.17–0.44, p < 0.001)	0.32 (0.18–0.54, p < 0.001)

Number in dataframe = 764, Number in model = 764, Missing = 0, AIC = 840.5, C-statistic = 0.714, H&L = Chi-sq(8) 6.08 (p = 0.639)
* One sex reported as *other* not presented in table.

Appendix Table 7. Univariable and multivariable odds ratios for factors associated with a positive SARS-CoV-2 virological test — Omicron BA.1 phase of transmission, 24 December 2021 to 30 April 2022

Variable	NAAT Negative, n (%)	NAAT Positive, n (%)	Univariate odds ratio	Multivariable odds ratio
Sex				
Female	235 (83.3)	47 (16.7)	*Ref*	*Ref*
Male	168 (92.3)	14 (7.7)	0.42 (0.21–0.76, p = 0.006)	0.42 (0.19–0.90, p = 0.030)
Age cat, years				
2–17	145 (98.6)	2 (1.4)	0.07 (0.01–0.23, p < 0.001)	0.66 (0.06–5.17, p = 0.704)
18–54	216 (83.4)	43 (16.6)	*Ref*	*Ref*
≥55	42 (72.4)	16 (27.6)	1.91 (0.97–3.67, p = 0.055)	1.48 (0.63–3.47, p = 0.365)
Vaccine doses				
None	135 (97.1)	4 (2.9)	*Ref*	*Ref*
One	25 (96.2)	1 (3.8)	1.35 (0.07–9.61, p = 0.792)	0.16 (0.01–2.31, p = 0.211)
Two	160 (78.8)	43 (21.2)	9.07 (3.56–30.71, p < 0.001)	1.03 (0.14–8.19, p = 0.975)
Three	82 (86.3)	13 (13.7)	5.35 (1.82–19.50, p = 0.004)	1.84 (0.25–15.16, p = 0.554)
Four	1 (100.0)	0 (0.0)	*NC*	*NC*
Anti–S titer (quantile)				
Q1	96 (82.8)	20 (17.2)	*Ref*	*Ref*
Q2	96 (82.8)	20 (17.2)	1.00 (0.50–1.98, p = 1.000)	0.69 (0.27–1.76, p = 0.442)
Q3	102 (87.9)	14 (12.1)	0.66 (0.31–1.37, p = 0.268)	0.46 (0.16–1.27, p = 0.141)
Q4	109 (94.0)	7 (6.0)	0.31 (0.12–0.73, p = 0.011)	0.14 (0.04–0.42, p = 0.001)

Number in dataframe = 464, Number in model = 464, Missing = 0, AIC = 237.8, C-statistic = 0.921, H&L = Chi-sq(8) 4.05 (p = 0.853).

Appendix Table 8. Univariable and multivariable odds ratios for factors associated with a positive SARS-CoV-2 virological test — Omicron BA.2, BA.4, BA.5 phase of transmission, 1 May 2022 to 17 August 2022

Variable	NAAT Negative, n (%)	NAAT Positive, n (%)	Univariate odds ratio	Multivariable odds ratio
Sex				
Female	211 (76.2)	66 (23.8)	*Ref*	*Ref*
Male	121 (81.2)	28 (18.8)	0.74 (0.45–1.20, p = 0.233)	0.80 (0.45–1.38, p = 0.429)
Age cat, years				
2–17	121 (88.3)	16 (11.7)	0.36 (0.19–0.64, p = 0.001)	0.33 (0.12–0.88, p = 0.030)
18–54	173 (73.3)	63 (26.7)	*Ref*	*Ref*
≥55	38 (71.7)	15 (28.3)	1.08 (0.54–2.07, p = 0.812)	1.18 (0.55–2.43, p = 0.668)
Vaccine doses				
None	109 (84.5)	20 (15.5)	*Ref*	*Ref*
One	15 (71.4)	6 (28.6)	2.18 (0.71–6.10, p = 0.150)	1.07 (0.24–4.40, p = 0.931)
Two	133 (74.3)	46 (25.7)	1.88 (1.07–3.43, p = 0.033)	1.09 (0.30–3.83, p = 0.894)
Three	71 (76.3)	22 (23.7)	1.69 (0.86–3.34, p = 0.128)	0.97 (0.29–3.26, p = 0.965)
Four	4 (100.0)	0 (0.0)	*NC*	*NC*
Anti–S titer (quantile)				
Q1	73 (68.2)	34 (31.8)	*Ref*	*Ref*
Q2	80 (74.8)	27 (25.2)	0.72 (0.40–1.31, p = 0.290)	0.59 (0.30–1.15, p = 0.123)
Q3	87 (82.1)	19 (17.9)	0.47 (0.24–0.88, p = 0.021)	0.30 (0.14–0.62, p = 0.001)
Q4	92 (86.8)	14 (13.2)	0.33 (0.16–0.64, p = 0.002)	0.24 (0.10–0.53, p = 0.001)

Number in dataframe = 426, Number in model = 426, Missing = 0, AIC = 412.2, C-statistic = 0.764, H&L = Chi-sq(8) 8.77 (p = 0.362).

Appendix Table 9. Multivariable odds ratios for a positive SARS-CoV-2 virological test by anti-S quartile and number of days from symptom onset to sample collection (N = 2,300)

Anti-S titer, quartile	DPSO 0–4 d (n = 1,541)	DPSO ≥4 d (n = 759)
Q1	*Ref*	*Ref*
Q2	0.56 (0.38–0.81, p = 0.002)	0.35 (0.19–0.63, p = 0.001)
Q3	0.32 (0.21–0.49, p < 0.001)	0.47 (0.23–0.93, p = 0.031)
Q4	0.21 (0.13–0.33, p < 0.001)	0.37 (0.17–0.79, p = 0.011)

Ref = reference. Odds ratios with 95% CIs calculated using binomial multivariable logistic regression models with data presented for log-adjusted anti-S titers stratified by quartile. Quartiles calculated using the *quantile* function in R. Model covariates include number of COVID-19 vaccine doses received, days since last COVID-19 vaccine dose, anti-S titers, sex, age, and month of sample collection.

Appendix Table 10. Anti-S levels by quartile and phase of predominant SARS-CoV-2 variant strain

Phase	Quartile	GMT, BAU/ml (955 CI)	Median titer, BAU/ml (Q1, Q3)
Pre-delta	Q1	1.4 (1.2, 1.6)	0.8 (0.4, 5.6)
	Q2	89 (77.7, 101.9)	91.7 (35.6, 186.8)
	Q3	1140.6 (1008, 1290.7)	1083 (854.6, 1486.5)
	Q4	12962.9 (7072.7, 23758.6)	8861.5 (5403.2, 23692.5)
Delta	Q1	1.3 (1, 1.8)	0.4 (0.4, 6.1)
	Q2	124.8 (111.8, 139.3)	127.8 (62.2, 242.2)
	Q3	1292.6 (1194.8, 1398.3)	1175.5 (784.7, 2061.8)
	Q4	14491.6 (12632.1, 16624.8)	11134 (6253.5, 30751.8)
Omicron (BA.1)	Q1	1.2 (0.8, 2)	0.4 (0.4, 9)
	Q2	138.2 (114.4, 167.1)	145.8 (73, 250)
	Q3	1603.8 (1463.2, 1758)	1689 (1041.8, 2621.5)
	Q4	11602.5 (10321.3, 13042.8)	9464 (6169.2, 20493.8)
Omicron (BA.2/4/5)	Q1	1.9 (1, 3.6)	0.4 (0.4, 19.3)
	Q2	131.8 (102.8, 168.9)	160.7 (67, 248.2)
	Q3	1716.3 (1579.7, 1864.8)	1832.5 (1152.8, 2881.2)
	Q4	7758.8 (7079.5, 8503.2)	6764 (4720, 10408)

Number of individuals per phase are 646, 764, 464, and 426 for pre-Delta, Delta, Omicron (BA.1), and Omicron (BA.2//4/5) respectively, with Quartiles calculated using the *quantile* function in R.

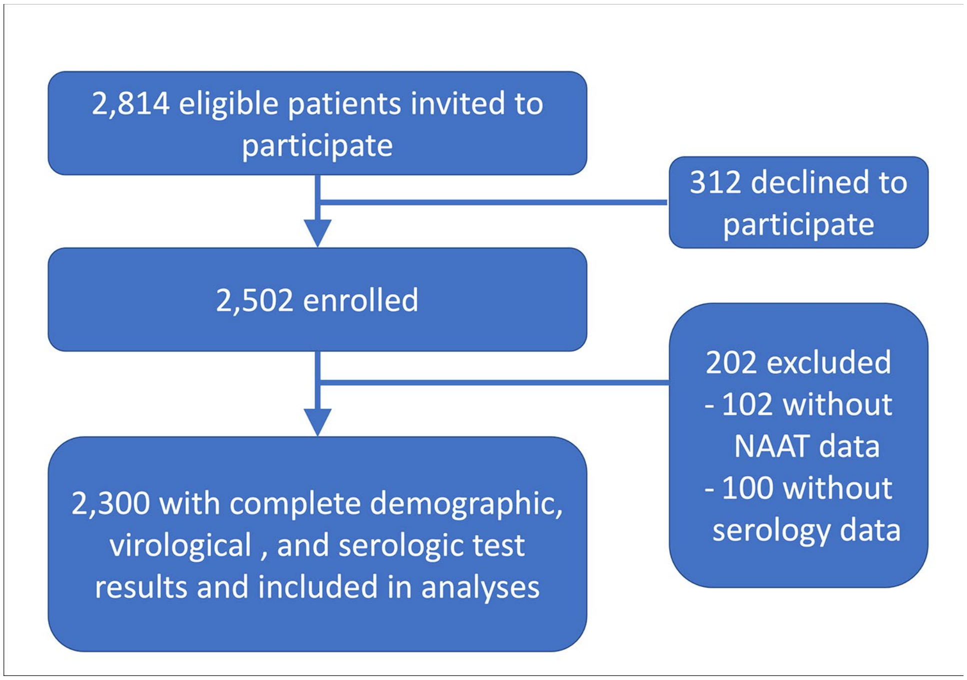

Appendix Figure 1. Study participant enrollment.

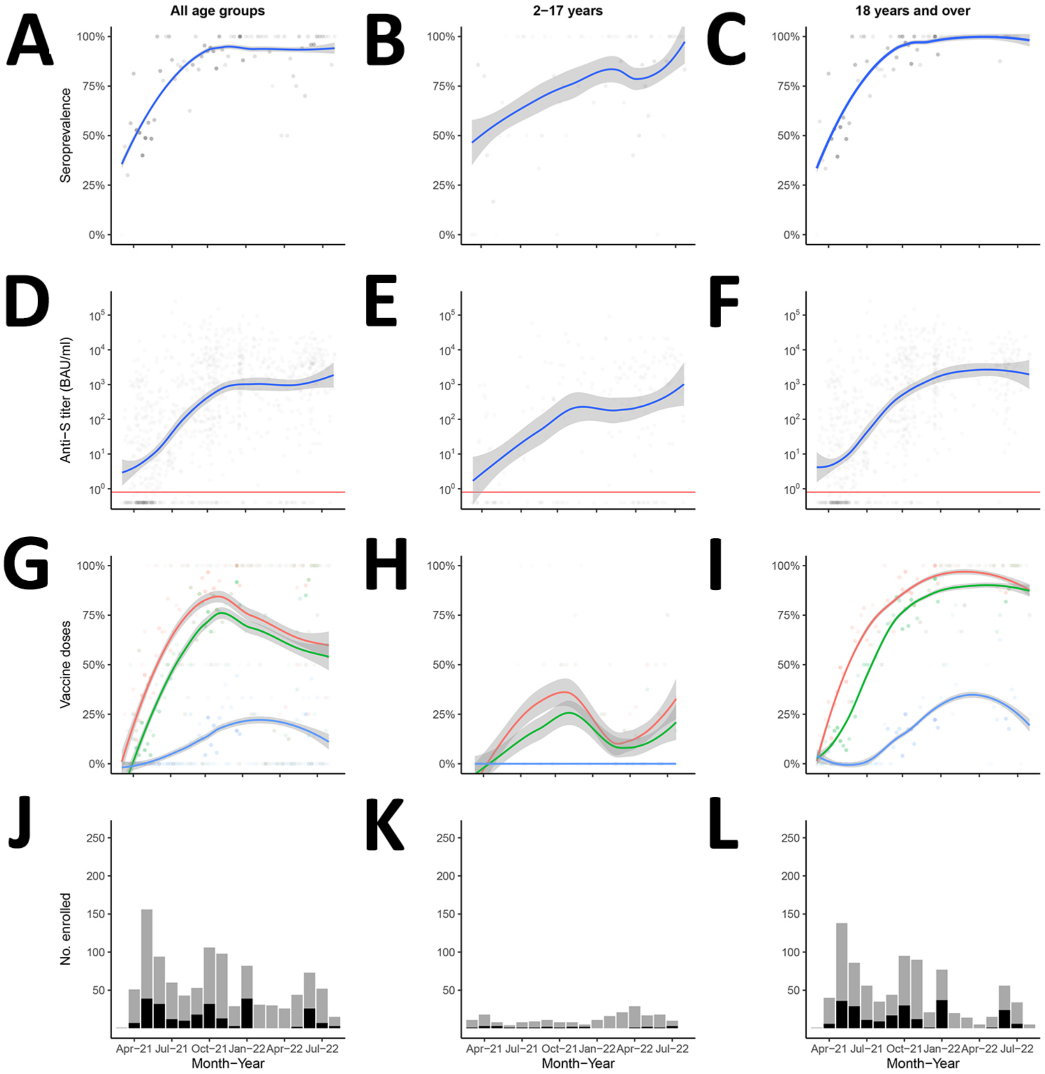

Appendix Figure 2. Anti-spike seroprevalence, titers, vaccine doses and participant enrollment by age group, Hospital Dr. Antonio Musa, San Pedro de Macoris, Dominican Republic, April 2021–August 2022 (n = 1,045).

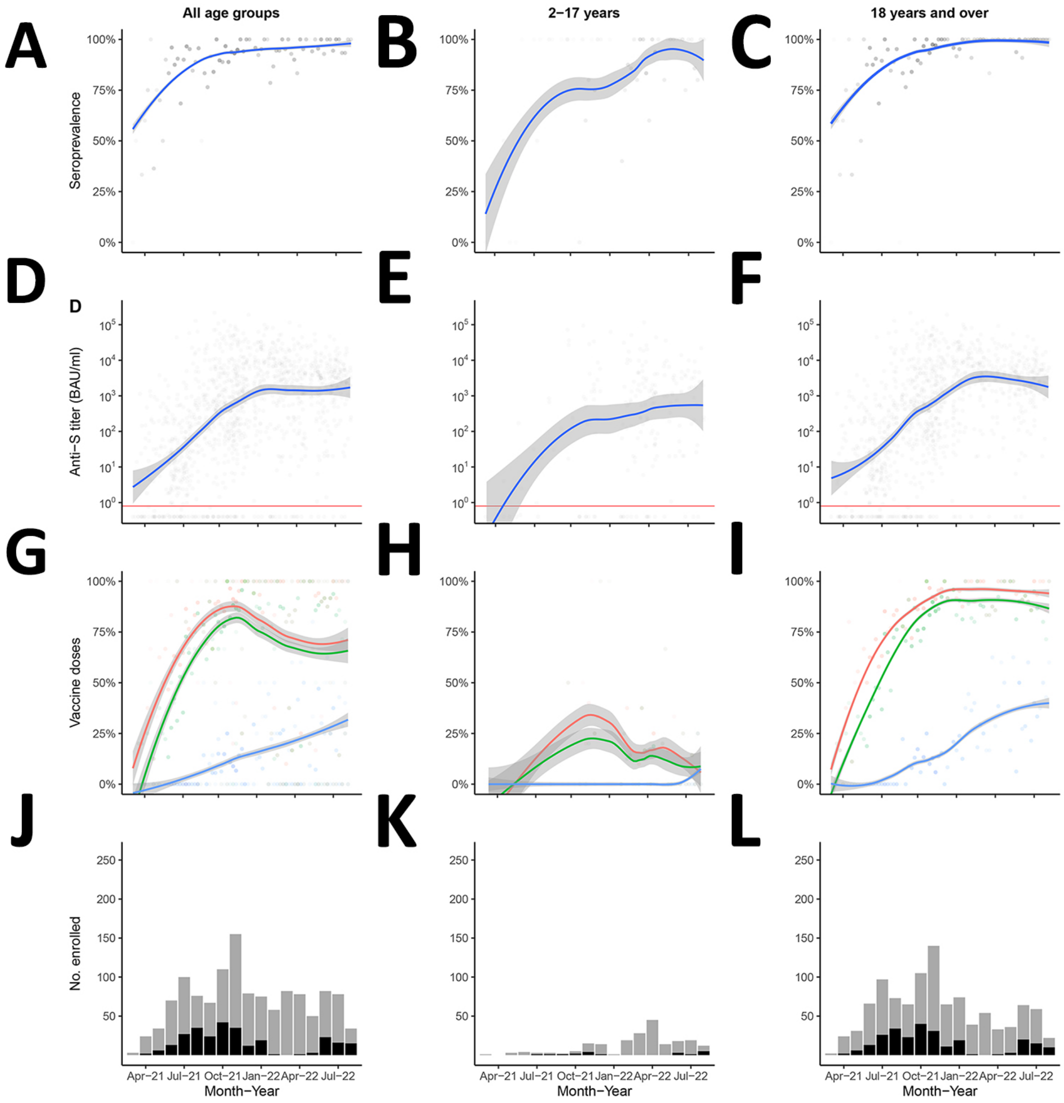

Appendix Figure 3. Anti-spike seroprevalence, titers, vaccine doses and participant enrollment by age group, Dr. Toribio Bencosme Hospital, Espaillat Province, Dominican Republic, April 2021–August 2022 (n = 1,255).

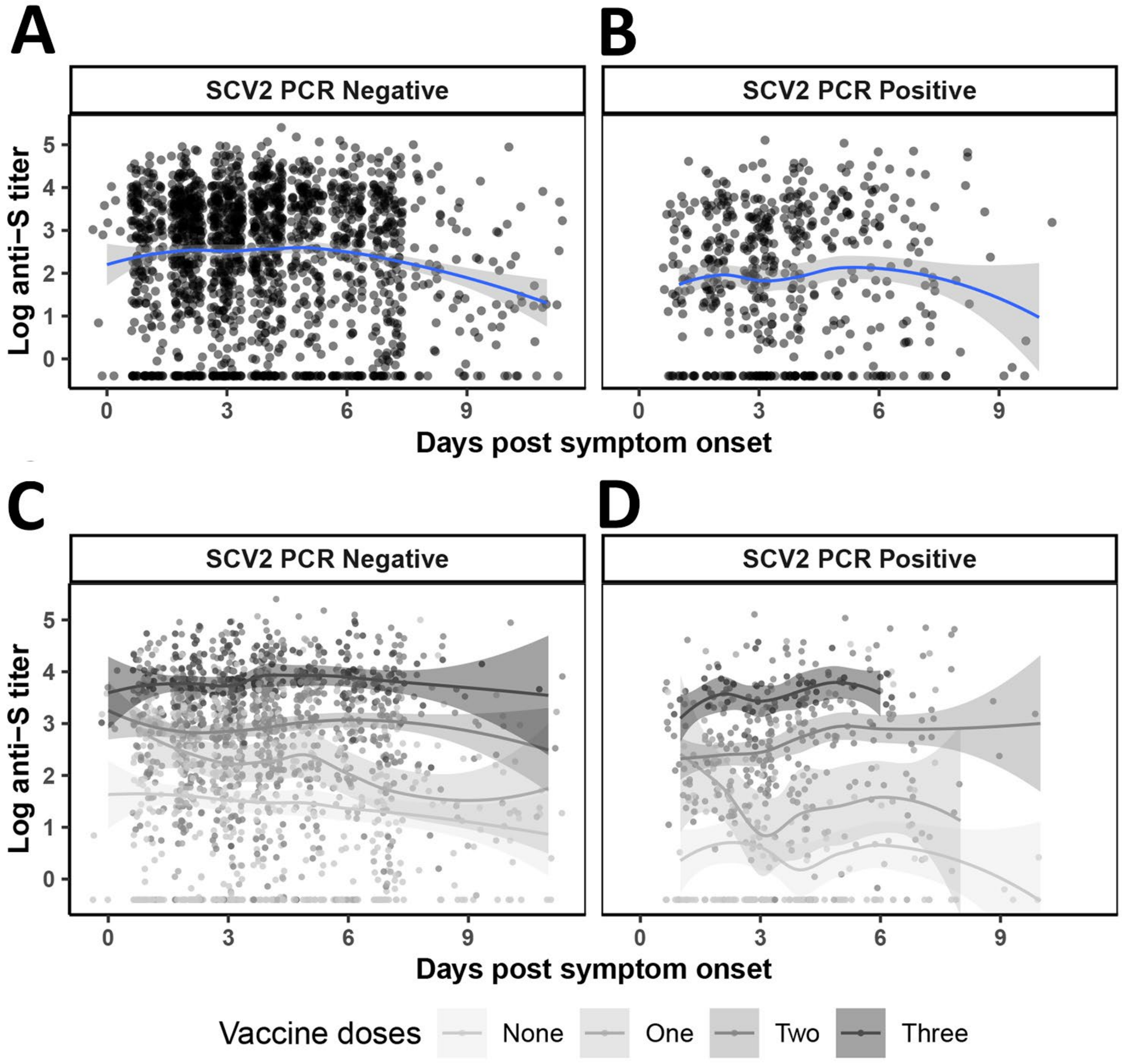

Appendix Figure 4. SARS-CoV-2 anti-S titers by days post-symptom onset, Dominican Republic, March 2021 - May 2022. Top plots represent anti-S titer on the y-axis and the number of days after symptom onset that the sample was collected on the x-axis, stratified by NAAT status. LOESS smoothed line and 95% CI marked with a blue line with gray shading respectively. Jittered dots represent individual study participants. No clear trend is observed. Bottom plots reflect plots above but stratified by number of COVID-19 vaccine doses received. A trend to an increase in titers is observed among NAAT positive individuals who received two or three vaccine doses, although the small number of participants makes clear interpretation challenging.

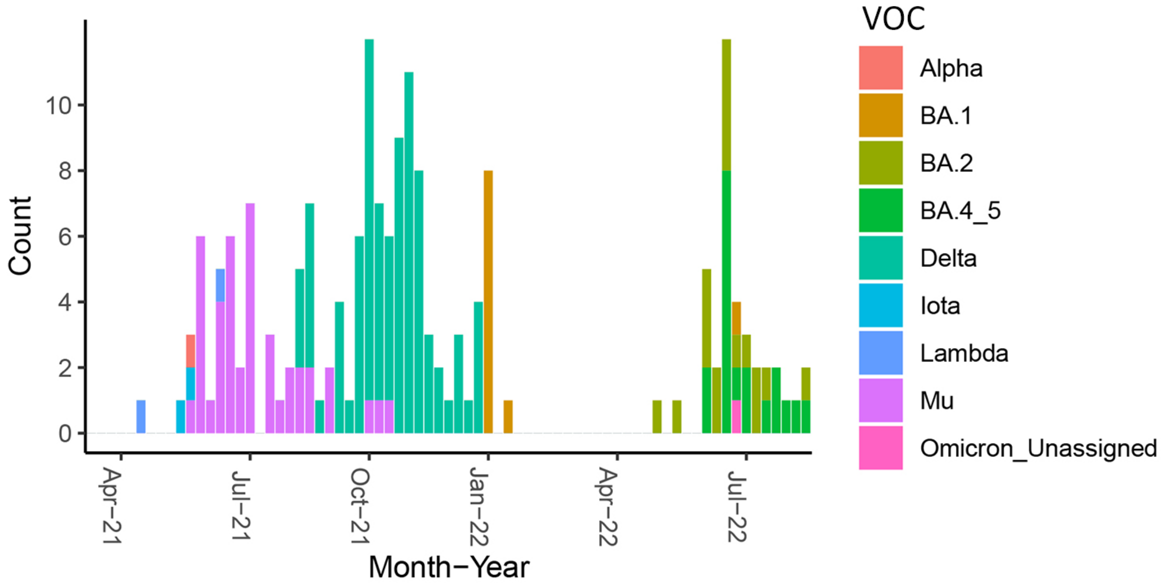

Appendix Figure 5. SARS-CoV-2 variant, Espaillat and San Pedro de Macoris, Dominican Republic, March 2021 - May 2022.
